# A Frequency Independent Framework for Synthesis of Programmable Non-reciprocal Networks

**DOI:** 10.1038/s41598-018-32898-x

**Published:** 2018-10-02

**Authors:** Ruochen Lu, Jack Krol, Liuqing Gao, Songbin Gong

**Affiliations:** University of Illinois at Urbana-Champaign, Urbana, United States

## Abstract

Passive and linear nonreciprocal networks at microwave frequencies hold great promises in enabling new front-end architectures for wireless communication systems. Their non-reciprocity has been achieved by disrupting the time-reversal symmetry using various forms of biasing schemes, but only over a limited frequency range. Here we demonstrate a framework for synthesizing theoretically frequency-independent multi-port nonreciprocal networks. The framework is highly expandable and can have an arbitrary number of ports while simultaneously sustaining balanced performance and providing unprecedented programmability of non-reciprocity. A 4-port circulator based on such a framework is implemented and tested to produce a broadband nonreciprocal performance from 10 MHz to 900 MHz with a temporal switching effort at 23.8 MHz. With the combination of broad bandwidth, low temporal effort, and high programmability, the framework could inspire new ways of implementing multiple input multiple output (MIMO) communication systems for 5G.

## Introduction

Microwave frequency nonreciprocal networks that bear non-reciprocal responses have long been sought after for a wide range of applications, including full-duplexing radios^1,2^ and quantum computing^3–5^. Most commonly utilized nonreciprocal multiport systems are isolators and circulators. Conventionally, non-reciprocity is obtained by magnetically biasing a ferrite material within which the electromagnetic wave propagates at different phase velocities in the opposite directions^6,7^. In a circular structure based on a material of such properties, constructive and destructive interference of the clockwise and counter-clockwise propagating waves can exist at different nodes around the circular resonator, thus establishing transmission and isolation through ports situated at these nodes.

Motivated by attaining non-reciprocity for more integrated RF and microwave applications, temporal modulations, applied to either reactive^8–10^ or conductive^11,12^ elements, have recently been explored to produce a momentum-biasing equivalent to the magnetic ones and break the reciprocity. These approaches all rely on wave interference or mode splitting caused by biasing in a resonant structure. In other words, the bandwidth over which their desirable non-reciprocal performance can be maintained is sensitive to phase delays between adjacent ports of the network. Although wide-band phase nonreciprocal gyrators^12^ can be engineered to enhance the bandwidth of such systems, this type of non-reciprocal devices is inherently frequency dependent. Moreover, demonstrations on temporally modulation enabled nonreciprocity so far are primarily two port gyrators^11,13^ and three port circulators^9^. Conceivably, both magnetic and temporal modulation based approaches can be expanded to a network with more ports by exploiting established circuit topologies or simply networking several three port circulators. However, the possibilities of reconfiguring the non-reciprocity in these approaches are limited. For instance, only a small subset of circulation sequences through all ports are accessible among all permutations, due to the limitations arising from their topologies and application of momentum biasing.

We show a framework for synthesizing a frequency independent and broadly programmable non-reciprocal network with an arbitrary number of ports (2*N*) using switches and an array of dispersionless delay lines. The generalized 2*N*-port framework can also be elegantly reduced to a 3-port or 2-port device with a more compact size and fewer switched delay lines than the sequentially switched delay lines^14^. This concept attains multi-port non-reciprocity by equally multiplexing the input signal onto *N* delay lines in the time domain and later aggregating the delayed signals off *N* delay lines consecutively at the intended port. The timing offset between switches addressing each port results in only one port receiving the signal at any given time from an excitation port. Unlike the abovementioned momentum biasing approaches, the non-reciprocal performance of our network is solely dependent on the time delays, instead of phase delays, and therefore is frequency independent. More impressively, the network has far more programmable states than any alternative reconfigurable non-reciprocity. Such programmability of nonreciprocity in a multi-port network will inspire new applications in multiple input multiple output (MIMO) communication systems.

## Results

**Figure 1:**
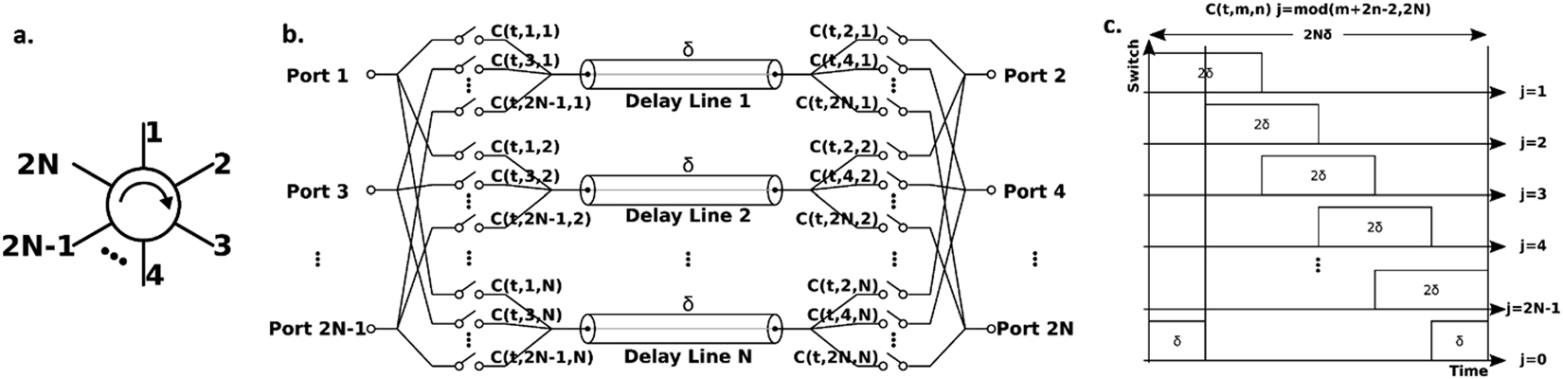
(**a**) Schematic symbol of a circulator of 2*N* ports with clockwise circulation. (**b**) Concept of the 2*N*-port non-reciprocal network. (**c**) Switch control waveforms applied to the network for producing the nonreciprocity.

Figure 1 shows the 2*N*-port framework consisting of 2*N* ports equally situated on both sides of *N* identical delay lines that each have a time delay of *δ*. On either side of the delay lines, each port is fanned out to connections with all delay lines through a single pole single throw (SPST) switch that presents open in the off-state and short in the on-state. Therefore, the composition of such a 2*N*-port network requires *N* delay lines and 2*N*^2^ SPSTs (or 2*N* SPNTs).

The clock signal for controlling each switch is denoted as *C*(*t*, *m*, *n*), where *t* is the time, *m* is the port number, *n* is the delay line number. All the clocks have a period of *2Nδ* and a duty cycle of *1/N*. Within the time range [0, *2Nδ*], the control signals can be represented as:

$$C(t,m,n)=\{\begin{array}{ll}{\rm{H}}[{\rm{t}}-({\rm{j}}-1){\rm{\delta }}]-{\rm{H}}[{\rm{t}}-({\rm{j}}+1){\rm{\delta }}] & {\rm{for}}\,{\rm{j}}\ne 0\\ {\rm{H}}[{\rm{t}}]-{\rm{H}}[{\rm{t}}-{\rm{\delta }}]+{\rm{H}}[{\rm{t}}-(2{\rm{N}}-1){\rm{\delta }}]-{\rm{H}}[{\rm{t}}-2{\rm{N}}{\rm{\delta }}] & {\rm{for}}\,{\rm{j}}=0\end{array}$$

where H is the Heaviside step function, and *j* is the remainder of the modulo operation.

$${\rm{j}}=\,{\rm{mod}}({\rm{m}}+2{\rm{n}}-2,2{\rm{N}})$$

*C*(*t*, *m*, *n*) is designed to turn on only one switch, among the switches connected to Port *m*, at any given time so that the signal is sequentially time-multiplexed onto the *N* delay lines. On the other side of delay lines, Port *m* + *1* is controlled by C(*t*, *m* + *1*, *n*), which is designed to be a time delayed version of C(*t*, *m*, *n*) with a timing offset of *δ* so that the signal, will be collected and de-multiplexed into Port *m* + *1*, after traversing *N* delay lines.

In the reverse path, signals fed into Port *m* + *1*, after being time multiplexed onto and traversing the delay lines, are subsequently rejected by port *m* because the switching control clocks, C(*t*, *m*, *n*), are the time advanced versions of C(*t*, *m* + *1*, *n*). In other words, all switches are turned off as the signal arrives Port *m* from Port *m* + *1*. On the other hand, switches on Port *m* + *2* are synchronized with the arrival of signals from Part *m* + *1* to aggregate them from the delay lines. The exception exists for Port 2*N*, to which the fed signals will be circulated to Port 1.

For a 2*N*-port network that consists of infinitely fast and lossless switches, lossless and dispersionless delay lines, and is addressed by ideal square wave control signals, infinitely large isolation, zero insertion loss, and zero return loss can be obtained. The perfectly synchronized time-domain multiplexing and de-multiplexing on the opposite ends of the *N* delay lines allow signal incident from Port *m* to exclusively be transmitted to Port *m* + *1*, while the energy leakage in the reverse order is completely forbidden.

**Figure 2:**
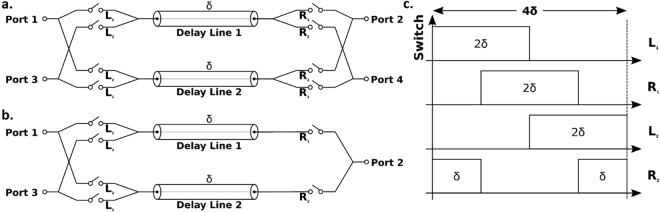
(**a**) Schematic of a 4-port circulator. (**b**) Schematic of a 3-port circulator reduced from a 4-port circulator. (**c**) Switch control waveforms for producing clockwise circulation (from Port 1 to 4).

Note that in our generalized framework, *N* has to be an even number as required by the symmetry of the network. For producing an odd number of ports, a network with an even number of ports can be reduced to have one less port by leaving one port open, which essentially eliminates *N* SPSTs. As an example seen in Fig. 2 (a), (b), a four-port network is reduced to a three-port circulator that is typically sought after for full-duplex radio applications.

### Four-port broadband circulator and experimental validation

To experimentally validate our framework, we choose to produce a four-port circulator based on the 2*N*-port framework with a frequency span from DC to 1 GHz. Figure 2 shows the schematic and control waveforms.

**Figure 3:**
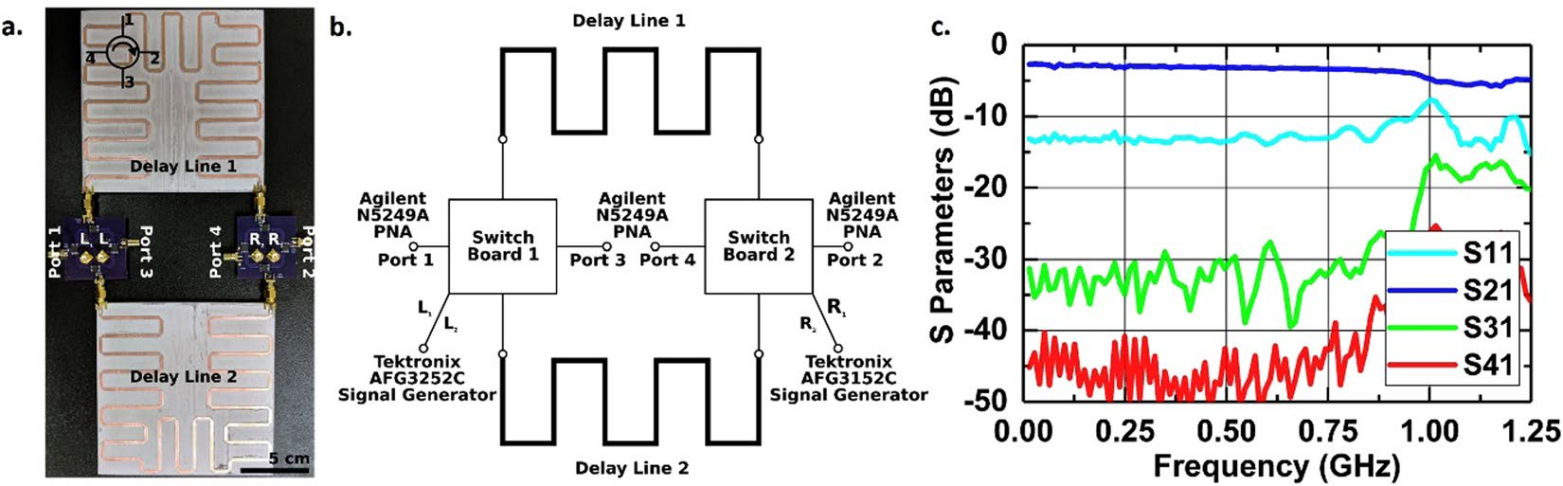
(**a**) Picture of the implemented 4-port circulator consisting of two switching boards and two microstrip delay lines. (**b**) System schematic of the 4-port circulator. (**c**) Simulated S-parameters of the 4-port circulator.

As seen in Fig. 3 (a), the prototype is implemented with connectorizied switching boards and delay line modules. Two delay line modules, with each end connected to a switching board, form the nonreciprocal network. We take the modular approach for experimental validation as it allows more nodes in the 4-port network to be experimentally observed for loss analysis.

Based on our design for a 4-port switching board, only two SPST series switches that present near open circuit to the input in the off-state is needed. In practice, open-reflective switches with a fast switching time are not commonly available. Alternatively, four short-reflective switches, minicircuit MSW 2–20+, are arranged in a lattice configuration (see supplementary materials) to produce the switching performance of two SPST open-reflective switches equivalently. MSW 2–20+ has a fast switching time of 2 ns, which minimizes the insertion loss due to switching. The delay line modules are implemented using Roger Duroid 6010.2LM boards with meandering microstrip structures to produce a total group delay of 10.5 ns with a slight dispersion that is less than 1 ns.

In operation, the switches are controlled by four clock signals that have a period of 42 ns, and a frequency of 23.8 MHz. The slightly increased delay is caused by the additional electrical lengths on the switching boards. The switches on the same side of the delay line are complementarily driven while the switches on the opposite ends of the same delay are driven with a timing offset of 10.5 ns to satisfy the modulation signal requirement shown in Fig. 2. The clock signals are generated by two synchronized dual-channel Tektronics arbitrary function generators and fed to the control ports on the switching boards.

Advanced Design System (ADS) is used for simulating the 4-port performance. The switches have a switching time of 2 ns, an on-state resistance of 3Ω, and an off-state resistance of 60 kΩ. The delay lines are represented by their S-parameter performance, which is modeled using ADS momentum. To extract the frequency domain response of the network, a series of time domain simulations with varying single tone inputs to Port 1 is performed before Fourier transform is performed to attain scattered power out of the other ports at the input frequency. As seen in Fig. 3 (c), the simulation shows broadband (up to 0.9 GHz) nonreciprocal performance. An insertion loss (IL) of 3 dB at low frequencies is caused by the non-ideal switch properties and the loss in the delay lines. High isolation over 30 dB is observed simultaneously. The performance degrades towards higher frequencies due to the additional loss in the delay lines.

The measurement of the 4-port network was done using a setup shown in Fig. 3 (b). The non-reciprocal network is tested with a 4-port Keysight PNA-X network analyzer. E-cal is performed to move the measurement reference planes to the connectors on the switch modules. 4-port S-parameters are subsequently characterized with an IF bandwidth of 1 kHz and a measured power level of −10 dBm.

**Figure 4:**
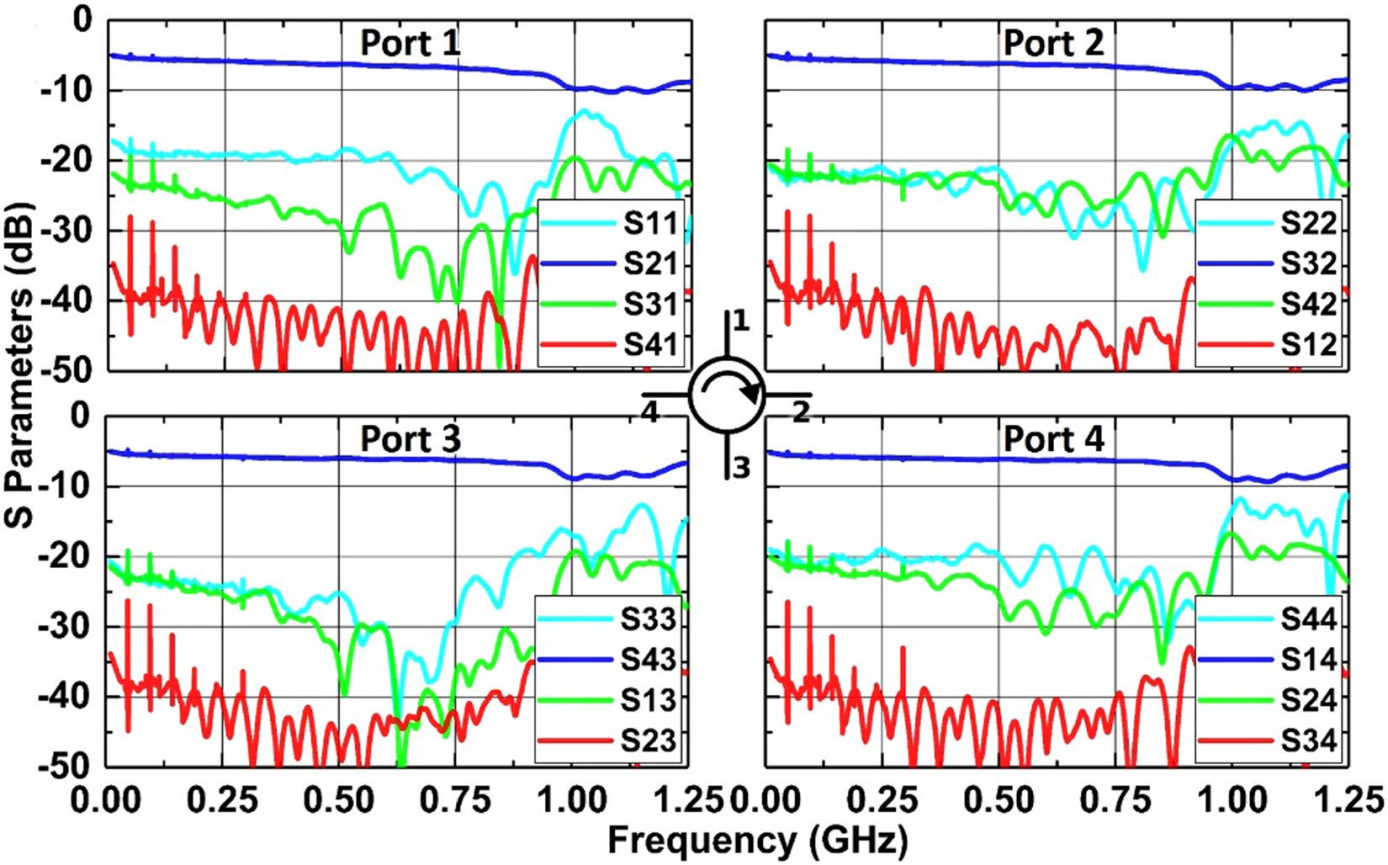
Measured S-parameter performance of the 4-port circulator.

As seen in Fig. 4, broadband non-reciprocal responses are obtained from 10 MHz to 0.9 GHz. A minimum IL of 5.1 dB is achieved at low frequencies. Isolations of 35 dB are measured between the adjacent ports and 20 dB between the diagonal ports. The 5.1 dB IL is collectively contributed by the static 2.1 dB IL from the 4 switches in the forward path (0.55 dB from each switch), the 0.8 dB loss caused by the switch rise time (more details in the supplementary materials), and the 2 dB IL from the interconnects. As the frequency increases to the self-resonance in the delay lines around 0.9 GHz, the IL and isolation performance gradually decay to 7.6 dB and 24 dB, respectively. The 7.6 dB IL is collectively contributed by the static IL from the 4 switches in the forward path (0.8 dB for each switch), the 0.8 dB loss caused by the switching rise time (more details in the supplementary materials), the 2 dB IL from the interconnects, and the 1.6 dB from the microstrip delay lines. The measured performance slightly deviates from the simulated results. The difference in the measured and simulated *S11* is due to our simplified switch model (more details in the supplementary materials). Although the model obtains the correct IL for transmission, it over-attributes the loss in the transmission to the return loss of the switch, instead of the dissipation in the switch. As a result, it overestimates the return loss of the whole system. The difference in *S21* at low frequencies is due to the additional 2 dB IL from the interconnects and multi-reflections between ports, while the slight difference in *S31* is caused by the weak signal leakage between ports of the switch board. The *S41* measurement matches simulation well. More details about the measured nonlinearity and intermodulation tones measured in the prototype are shown in the supplementary materials.

## Discussion

### Frequency independent performance

As discussed earlier, the frequency independent performance of nonreciprocity is the outcome of the perfect synchronization of time-domain multiplexing and delays in the forward path, and the complete off-synchronization between them in the backward route. We recognize that some causes in practice can compromise the frequency independent performance and yield a broadband performance instead. For instance, the electromagnetic delay lines typically exhibit dispersion, which causes the synchronization between switching and delays to degrade as the operating frequency moves away from the design center frequency. To reduce the size, delay lines based on slow-wave or meandering structures often have a cut off frequency that also limits the BW of the nonreciprocal network. Other types of delay lines with smaller sizes, e.g., acoustic delay lines^15^, usually have passbands over which low insertion loss and constant group delay can be maintained. Nonetheless, with our frequency independent nonreciprocal framework as the basis, the bandwidth over which non-reciprocity is enabled should be only limited by the components chosen for implementation, but not by the framework itself.

It is worth noting that the frequency independent performance is not dependent on the temporal effort applied in the system. Unlike the momentum biasing approaches where the bandwidth of nonreciprocity is fundamentally limited by the modulation frequency used to produce momentum biasing^16^, the switching frequency in our framework is only set by the time delay length imposed by the delay lines. Provided with low loss delay lines to render long group delays, one can reduce the switching frequency to a mere fraction of the non-reciprocal bandwidth (e.g., 23.8 MHz switching frequency for maintaining a nonreciprocal bandwidth of 900 MHz in our case). The low-frequency control signal consequently gives rise to simpler and lower cost clock generation, less phase delay in clock signal fanout, and minimized overall temporal effort. One caveat in operating our framework lies in the resulting group delays between ports, which is longer than those of ferrite circulators. Therefore, such systems might not be a good fit for timing-sensitive applications (e.g., radar front-ends).

### Network expandability without compromising performance and symmetry

Expanding a momentum-biased three port circulator into an *N*-port circulator is a non-trivial task. Merely adding more folds of symmetry in the structure will not produce unilateral circulation. In other words, the excitation at one port will be nonreciprocally received at more than one port. A typical way to attain nonreciprocal networks with more ports using momentum-biased devices is to network 3-port circulators in various manners, such as the method reported for creating macroscale topological materials^17^. With each added circulator in the network, the number of ports in the network can only be increased by one, thus suggesting a substantial cost in component counts and clock feeds for constructing multi-port nonreciprocal networks beyond three ports. Additionally, networking 3-port circulators often breaks the network structural symmetry and creates unbalanced paths between ports. Consequently, a higher IL is expected for paths that require the signal to traverse more in the composed multi-port network to reach destination ports.

For the even-port operation in our time-multiplexed framework, one can add two more ports to the network with each added delay line, which compares favorably against the network expansion via interconnecting 3-port circulators. For networks with an odd number of ports, the cost of expansion is the same, except for adding the last port, which requires a delay line for its own. More importantly and more advantageously in our framework, all transmission paths are balanced with the same IL and delay regardless of the number of ports. Thus, the 2*N*-network maintains *N* folds of symmetry in both the structural design and performance.

### Programmability of nonreciprocity with a rich space of permutations

Enabling programmable RF circuits has been the holy-grail problem for designing highly adaptive RF systems in the past decade, focusing primarily on either passive reciprocal networks, such as filters^18–21^, antenna tuners^22^, and phase shifters^23^, or active/nonreciprocal circuits, such as amplifiers^24^. Programmability of passive non-reciprocity has rarely been visited even though the current carrier aggregated communication systems can significantly benefit from programmable non-reciprocity in front-ends^25^. Temporal modulated non-reciprocal systems have recently revived the hope for achieving such programmability without compromising other relevant performance specifications.

**Figure 5:**
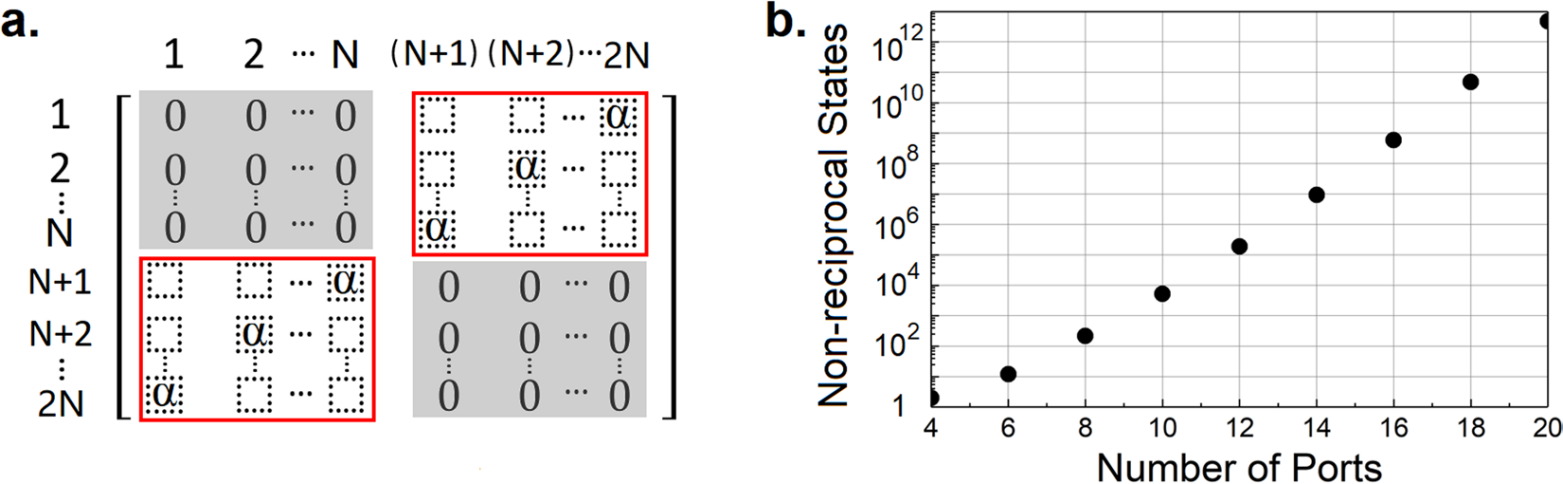
(**a**) Accessible and forbidden regions of the programmable S-Matrix. (**b**) Programmable non-reciprocal states as a function of the number of ports.

Our framework is readily programmable by first re-shuffling the clock waveforms applied to the switches on one side of the delay lines and then adjusting the clocks on the other side accordingly. Through this practice, any port on one side of the delay lines can be configured to circulate to any port on the other side of the delay lines, thus allowing for a rich space of non-reciprocal states. The accessible states for the 2*N*-port nonreciprocal network can be studied as S-matrix permutations with the only limitation that circulation between ports on the same side of the delay lines cannot be established. Therefore, assuming all ports are matched, the components in the shaded regions of the S-matrix, seen in Fig. 5 (a), are inaccessible for programming. On the other hand, assuming the network is lossless and S-matrix is unitary, the sub-matrices outlined by the red boxes in Fig. 5 (a) have a single complex component in each row and column. Provided that the implementation is balanced with identical switches on both sides of the identical delay lines, these complex components are identical with a magnitude of 1 and are denoted as α. Note that the programming of the network changes neither the structural nor the performance symmetry. In other words, the programming does not change the value of α in the S-matrix.

To determine the number of programmable non-reciprocal states, we can first populate the top right sub-matrix, referred to as sub-matrix A onward, with allowed permutations, which is *N*!. With each permutation of A, we can then exam the allowed permutations of sub-matrix B in the lower left quarter. Due to non-reciprocity of the network (*S*_*ij*_ ≠ *S*_*ji*_), *N* components are determined as 0 in B for a given permutation of A. Consequently, the number of ways to populate B for a given A is given by:

$$\begin{array}{rcl}P(N\times N,N) & = & N!-{C}_{N}^{1}\times (N-1)!+{C}_{N}^{2}\times (N-2)!-{C}_{N}^{3}\times (N-3)!+\ldots \\  &  & {(-1)}^{N-1}\times {C}_{N}^{N-1}\times (1!)+{(-1)}^{N}\times {C}_{N}^{N}\end{array}$$

Thus, the number of nonreciprocal states, *Ω* for a 2*N*-port network is:

$${\rm{\Omega }}(2N)=(N!)\times {\rm{P}}(N\times N,N)$$

As seen in Fig. 5 (b), this represents an exponential growth of programmable non-reciprocal states as the number of ports increases.

## Method

The loss in the system can be understood with an analytical approach focusing on the switching loss, which is defined as the IL caused by the switching process. Thus, when analyzing switching loss, the delay lines are modeled as lossless and perfectly matched transmission lines. Fundamentally, the switching loss is the result of momentarily losing the signal during the switching from one delay line to another. Such a loss is inevitable using switches with small but not zero switch-on and switch-off time. The IL due to switching is determined by how much the signal is lost proportionally over time and thus related to the ratio of switching time (*t*_*s*_) to delay time (*δ*). The switches are represented as time-varying resistances (*R*_*switch*_) during switching on and off periods. They linearly change resistances from an off-state resistance (*R*_*off*_) to an on-state resistance (*R*_*on*_) over a switching period (*t*_*s*_) upon the application of control waveforms, which are assumed to be perfect square waves with 50% duty cycle. In a *2δ* period, *R*_*switch*_ can be described as:

$${{\rm{R}}}_{{\rm{switch}}}({\rm{t}})=\,\{\begin{array}{ll}{{\rm{R}}}_{{\rm{off}}}+({{\rm{R}}}_{{\rm{on}}}-{{\rm{R}}}_{{\rm{off}}})\cdot \frac{{\rm{t}}}{{{\rm{t}}}_{{\rm{s}}}} & \mathrm{for}\,0 < t\le {t}_{s}\\ {{\rm{R}}}_{{\rm{on}}}\, & {\mathrm{for}t}_{{\rm{s}}} < t\le 2{\rm{\delta }}-{t}_{s}\\ {{\rm{R}}}_{{\rm{on}}}+({{\rm{R}}}_{{\rm{off}}}-{{\rm{R}}}_{{\rm{on}}})\cdot \frac{{\rm{t}}-2{\rm{\delta }}+{{\rm{t}}}_{{\rm{s}}}}{{{\rm{t}}}_{{\rm{s}}}}\, & \mathrm{for}\,2{\rm{\delta }}-{t}_{s} < t\le 2{\rm{\delta }}\end{array}$$

Consider the upper line in Fig. 2 (a), the input signal from 0 <* t* < *t*_*s*_ experiences a time-varying transmission coefficient of h(*t*) when transmitting through the switch controlled by L_1_. Then, this signal is delayed by *δ*, and from *δ* < *t* < *δ* *+* *t*_*s*_ the signal experiences a transmission of h(*t* − *δ*) when transmitting through the switch controlled by R_1_. Given that switching time (*t*_*s*_) is smaller than *δ*, h(*t*) can be described as:

$$h({\rm{t}})=\frac{2{Z}_{0}}{{R}_{switch}(t)+2{Z}_{0}\,}{\rm{for}}\,0 < t\le 2{\rm{\delta }}$$

The transfer function, between Port 1 to 2 as seen in Fig. 2 (a), is given as:

$${H}_{sys}(\omega )=H(\omega )\cdot {e}^{i\delta \omega }\cdot H(\omega -\delta )=H{(\omega )}^{2}$$

where H(*ω*) is the Fourier transform of h(*t*). It is noteworthy that when *t*_*s*_ > 0, the system transfer function has components other than the DC component. It implies that the non-ideal switching produces signals at frequencies other than the input signal (e.g., the carrier frequency), which is another interpretation of the switching loss. Also, insertion loss is also introduced by *R*_*on*_ and *R*_*off*_. Thus, the total IL between ports can be described as:

$$IL=-\,20\,{\mathrm{log}}_{10}({v}_{out}/{v}_{in})=-\,20\,{\mathrm{log}}_{10}{H}_{sys}(0)$$

**Figure 6:**
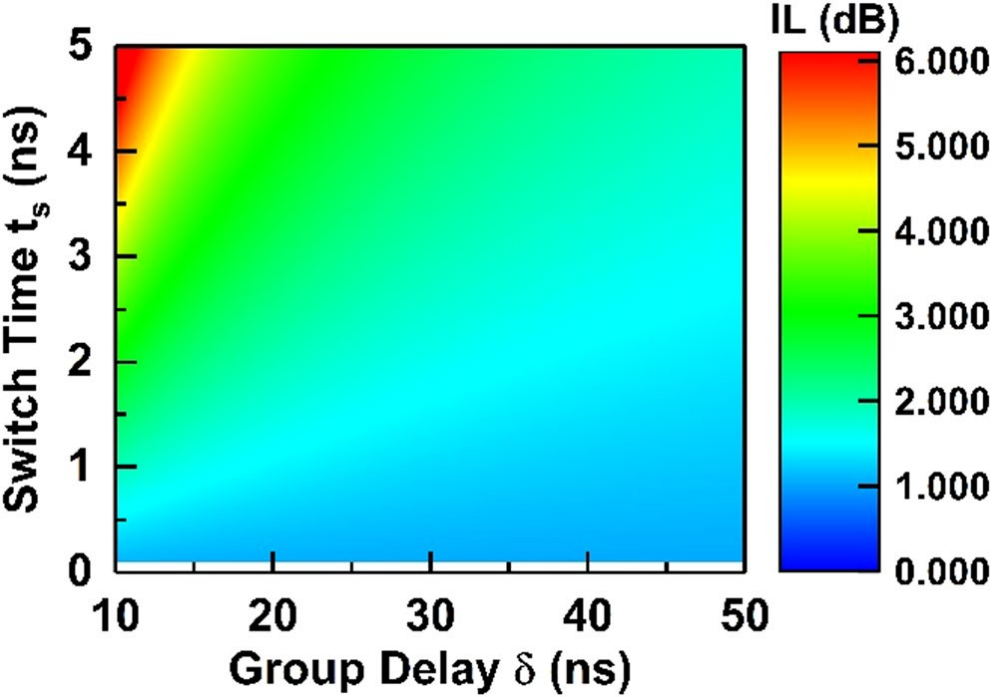
Dependence of switching loss on switching time and group delay.

Based on the analytical closed-form expression of switching loss as a function of *t*_*s*_ and *δ*, a 2D contour plot of switching loss with the switching time varying from 0 to 5 ns, and the group delay of the delay lines varying from 10 to 50 ns is plotted in Fig. 6. An *R*_*on*_ of 6 Ω and an* R*_*off*_ of 120 kΩ are assumed for the switches used in the implementation. For our specific implementation of 10.5 ns group delay and 2 ns switching rise time, the calculated IL is around 2.8 dB, which matches the measurement.

## Electronic supplementary material

Supplementary Material

## Data Availability

All relevant data is available upon request.
