# Supplementary material for: A Frequency Independent Framework for Synthesis of Programmable Non-reciprocal Networks
Source: Sci Rep. 2018 Oct 2;8:14655. doi: 10.1038/s41598-018-32898-x (PMC6168549; doi:10.1038/s41598-018-32898-x)
Supplement: Supplementary file 1 — Supplementary Material [file 41598_2018_32898_MOESM1_ESM.pdf]

Frequency Independent Framework for Synthesis of Programmable Non-reciprocal Networks

Ruochen Lu[1], John Krol[1], Liuqing Gao[1], and Songbin Gong*[1]

[1]University of Illinois at Urbana Champaign

* Correspondence to songbin@illinois.edu

Supplementary Materials:

1. Switch module implementation and control waveforms

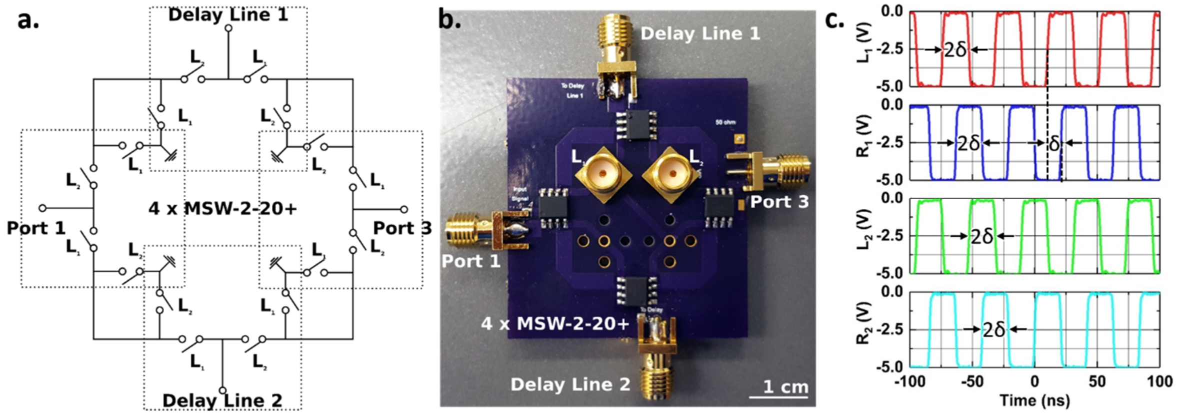

Fig. 1. Switch module design. (a) Schematic of the switch module. (b) Implemented switch module with interfaces labeled. (c) Control signals applied for system level measurement and produced by AFGs.

In our 4-port circulator implementation, two switch modules and two delay lines are required. The switch modules are identical, but 180° rotated from each other in the system assembly. For simplicity, the first switch module (with Port 1 and Port 3) will be discussed in details here.

The schematic of the switch module is shown in Fig. 1 (a). Each switch module consists of four single-pole, double-throw switches (SPDT, MSW-2-20+) that are interconnected between themselves and with two ports for the application of control signals (L_1 and L_2, 180° phase offset square waves), and four ports for routing the RF signals (Port 1, Port 3, Delay line 1, and Delay line 2). Different from the original schematic built on single-pole, single-throw (SPST) switches, two SPDT switches are connected in series to represent one SPST switch due to the lack of off-the-shelf open reflective SPST switches. In operation, due to the 180° phase offset, incident RF signals from Port 1 are sent to either Delay line 1 or Delay line 2, while incident RF signals from Port 3 are sent to the other delay line. The redundant switches connected to ground guarantee great isolation between Ports 1 and three due to additional reflection induced by shorting to ground.

The switch module is assembled on a 44.5-mm-wide, 1.6-mm-thick FR-4 printed circuit board (PCB), as shown in Fig. 1 (b). Two Amphenol ACX1230-ND SMA adapters are used for the control signal ports, while four Amphenol ACX 1652-ND SMA adapters are chosen for the RF signal ports. SMA types are selected for easier integration of the circulator system. The SMAs for control signals are placed near the center of the board to minimize the undesired phase difference between control signals applied to switches, and to reduce the video leakage of the control signals into the RF paths. The dimensions of RF signal interconnections between switches have been optimized to attain a low insertion loss for the switch modules.

As seen in Fig. 1c, the control signals are generated by two synchronized Tektronix AFG3000 arbitrary function generators (AFGs). 23.8 MHz square waves with an amplitude of 0 V to -5 V are generated in accordance with the delay δ (10.5 ns) imposed by the delay lines in the system. The rise time of each signal is 2 ns. The control signals, L_1 and L_2 or R_1 and R_2, are 180° offset in phase, while L_1 and R_1, as well as L_2

and R_2 , is 90° offset in phase. The loss caused by the switching modules have been experimentally identified with a Keysight N5249A PNA. The loss from port 1 to either delay line 1 or 2, when the switches at Port 1 are constantly turned on, is characterized to be 1.1 dB at 10 MHz. The loss is mainly from the IL of the switches (0.5 dB each). When the time-varying control signals as seen in Fig. 1 (c) is applied to the switches, the loss from port to delay lines is increased to 2.5 dB at 10 MHz. The measured loss agrees with the analysis of the switching loss shown in Fig. 6.

2. Delay line implementation and characterization

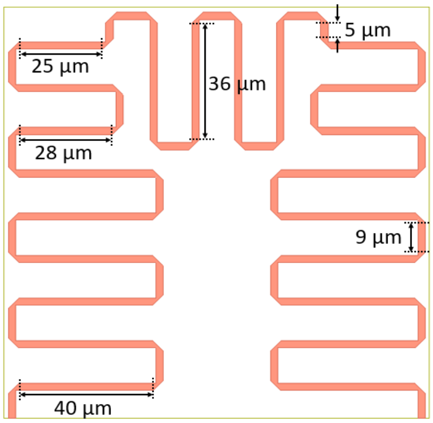

Fig. 2. Layout of the microstrip delay Line.

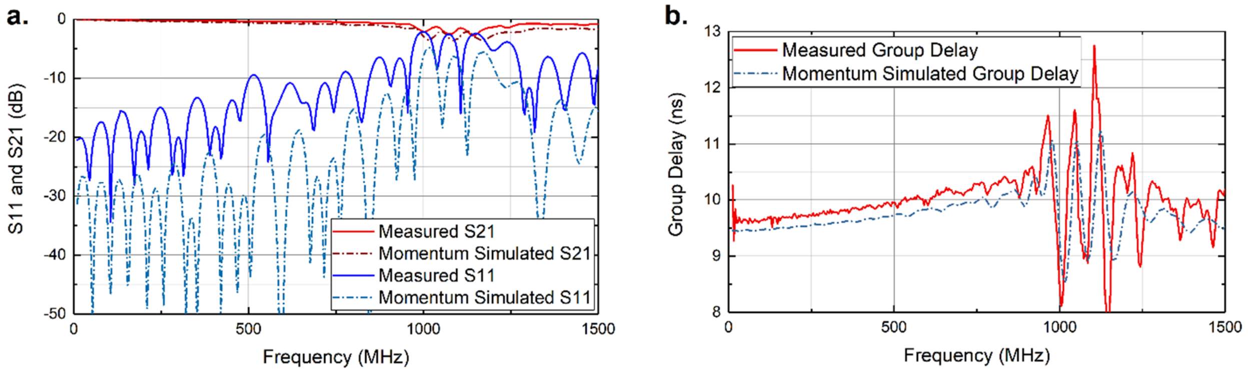

Fig. 3. Measurement and Momentum Simulation of S11, S21, and group delay of the microstrip delay line.

The delay lines are implemented based microstrip transmission lines on a Duroid board (6010.2LM) with a total length of 1071 mm. The substrate has a dielectric constant of 10.2 and a low loss tangent of 0.0023 up to 10 GHz. The large dielectric constant serves to minimize the phase velocity of wave propagation in the microstrip, and hence reduces the size for a given group delay. As seen in Fig. 2, a meandering layout with optimized bends, is adopted to reduce the size further and remove unwanted parasitic resonances below 1 GHz. The coupling between adjacent sections of meandering microstrip has been optimized to minimize unwanted ripple response in the group delay and insertion loss below 1 GHz. Optimization and modeling are done with ADS momentum.

The fabricated delay lines are tested before they are assembled with the switching modules. The measurement reference planes are moved to the SMA connectors in calibration. The measurement results are shown in Fig. 3, in comparison with the simulated results. The measurement results exhibit reasonable

agreement with Momentum simulation in terms of IL and group delay. An insertion loss of less than -2 dB is obtained, for a group delay around 10 ns below 1 GHz. Therefore, the insertion loss per unit delay is better than -0.06 dB/ns below 1 GHz. The measured return loss is higher than the simulated one. This is expected as the simulation does not include the effect of the SMA connectors. As predicted by simulation, the microstrip delay lines show worst return loss at 1 GHz and slight dispersion below 1 GHz, due to unwanted reflections in the adopted meandering layout.

3. Intermodulation in the system

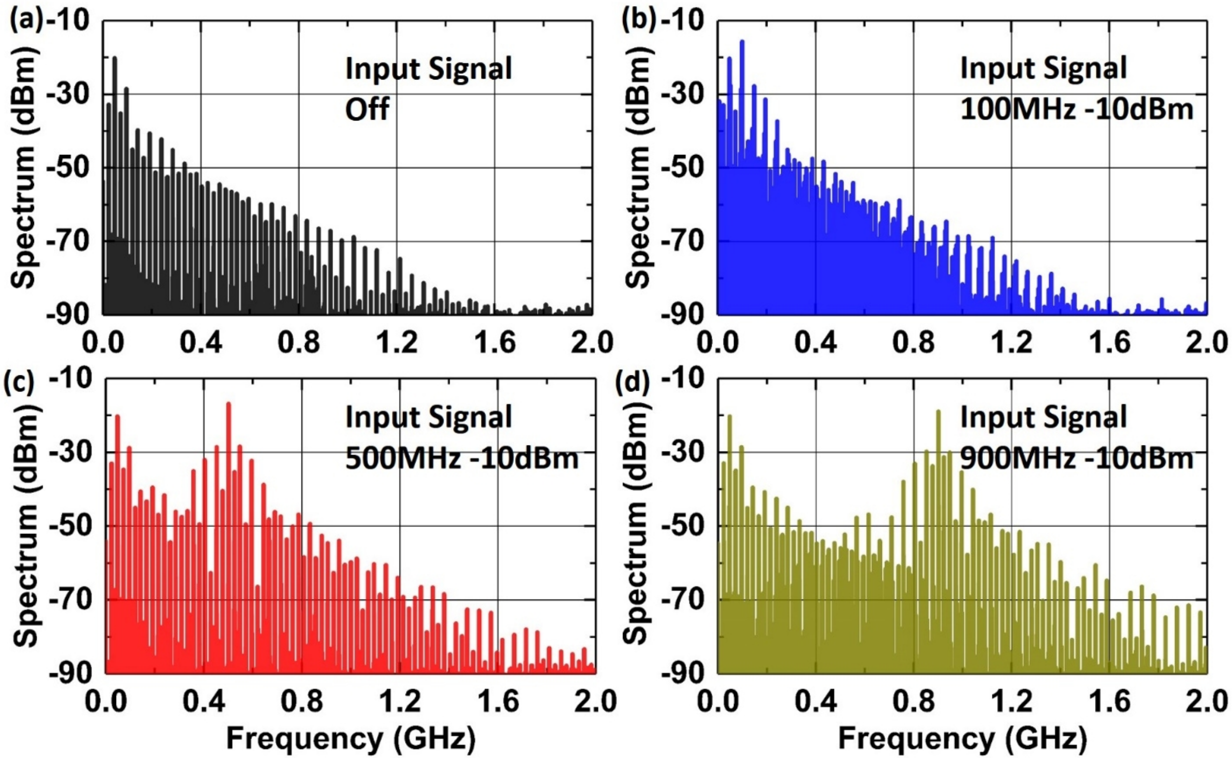

Fig. 4. Measured output spectrum at Port 2, when (a) a matched termination is connected to port 1, (b) 100 MHz, (c) 500 MHz, (d) 900 MHz signals at -10 dBm power level are sent to Port 1. Port 3 and 4 are terminated with matched termination.

Ideally, in our proposed frequency-independent nonreciprocal network, the output spectrum of different ports only contains the signal at the same frequency as the incident signal. However, two types of undesired tones exist in our prototype, namely the control signal leaked from the switching inputs and the intermodulation tones between the signal and the control signal caused by the switch rise-up time.

In order to identify the control signal leakage from the switches into the signal paths, the output spectrum is measured with an Agilent E4445A spectrum analyzer at Port 2 when Ports 1, 3, and 4 are terminated with matched terminations. As seen in Fig. 4 a., all the tones in the spectrum are caused by the leakage of control signals. The frequency spacing between adjacent tones is 23.8 MHz, which matches the frequency of the square-wave control signal. In our future work, the control signal leakage to the output spectrum can be significantly mitigated by using better switching modules (e.g., SOI switches) for better isolation and less crosstalk.

The first order intermodulation tones between the signal and the control signal are measured to be around 12 dBc from the main tone, as shown in Fig. 5 b-d. The spectrum is measured with an Agilent E4445A spectrum analyzer at Port 2 when -10 dBm signals at different frequencies are sent into Port 1 with Ports 3 and 4 matched. These tones are produced due to the switching rise and fall time, which can be shown with an approach similar to that in the Method Section. These intermodulation tones can be significantly suppressed when the ratio of switching time to group delay is sufficiently large. This requires either better switches with a shorter switching time[1] or longer group delays, which can be achieved by using either slow-wave structures[2] or acoustic delay lines[3].

4. Nonlinearity in the Implemented 4-port Nonreciprocal Network

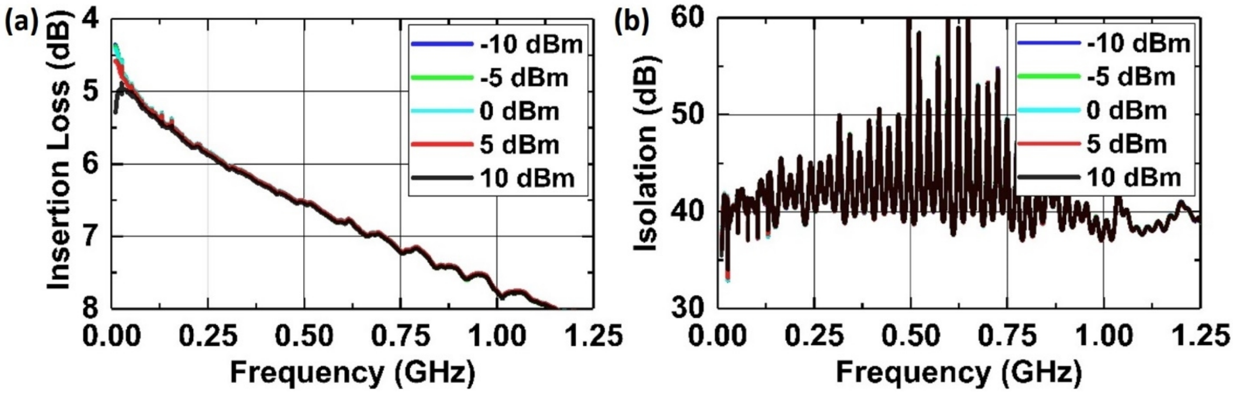

Fig. 5. Measured S-parameters at different power levels for both (a) transmission path S21 and (b) isolation path S12.

The nonlinearity is measured with Keysight PNA-X network analyzer at different power levels from -10 dBm to 10 dBm. Measured S-parameters of the transmission (S21) and isolation (S12) are shown in Fig. 5. The network shows good linearity up to 5 dBm. The 1-dB compression point of the transmission path in the system is around 10 dBm. The bottleneck of the linearity in our demonstrated prototype is the linearity of the switches (MSW-2-20), which indicates the maximum input power level of an individual switch as 11 dBm when used as a modulator.

References:

1. Ishida, H. *et al.* A high-power RF switch IC using AlGaN/GaN HFETs with single-stage configuration. *IEEE Trans. Electron Devices* **52,** 1893–1899 (2005).

2. Yang, F.-R., Qian, Y., Coccioli, R. & Itoh, T. A novel low-loss slow-wave microstrip structure. *IEEE Microw. Guid. Wave Lett.* **8,** 372–374 (1998).

3. Manzaneque, T., Lu, R., Yang, Y. & Gong, S. Lithium Niobate MEMS Chirp Compressors for Near Zero Power Wake-Up Radios. *J. Microelectromechanical Syst.* (2017). doi:10.1109/JMEMS.2017.2750176
